# Safety of Lavender Oil-Loaded Niosomes for In Vitro Culture and Biomedical Applications

**DOI:** 10.3390/nano12121999

**Published:** 2022-06-10

**Authors:** Janice de M. V. Vilela, Saeid Moghassemi, Arezoo Dadashzadeh, Marie-Madeleine Dolmans, Ricardo B. Azevedo, Christiani A. Amorim

**Affiliations:** 1 Pôle de Recherche en Gynécologie, Institut de Recherche Expérimentale et Clinique, Université Catholique de Louvain, 1200 Brussels, Belgium; janice.vilela@uclouvain.be (J.d.M.V.V.); saeid.moghassemi@uclouvain.be (S.M.); arezoo.dadashzadeh@uclouvain.be (A.D.); marie-madeleine.dolmans@uclouvain.be (M.-M.D.); 2 Gynecology and Andrology Department, Cliniques Universitaires Saint-Luc, 1200 Brussels, Belgium; 3 Laboratory of Nanobiotechnology, Department of Genetics and Morphology, Institute of Biological Sciences, University of Brasília, Brasília 70910-900, Brazil; razevedo@unb.br

**Keywords:** drug delivery systems, niosome, essential oils, regenerative medicine

## Abstract

(1) Background: Essential oils have long been used as therapeutic agents. Lavender (*Lavandula angustifolia*) oil (LO) is an antispasmodic, anticonvulsant, relaxant, painkilling, and antimicrobial essential oil investigated as a natural substance for biomedical therapies. Nanoparticles have shown significant promise in improving drug delivery and efficacy. Considering these benefits, the aim of this study was to evaluate the toxicity of LO and lavender oil niosomes (LONs) in stem cells and myofibroblast models cultured in vitro. (2) Methods: Adipose tissue-derived stem cells and myometrial cells were cultured with LO or LONs at different concentrations (0, 0.016%, 0.031%, and 0.063%) and toxicity was evaluated with PrestoBlue™ and live/dead assay using calcein and ethidium homodimer. (3) Results: Cell viability was similar to controls in all groups, except in 0.063% LO for myometrial cells, which showed lower viability than the control medium. (4) Conclusion: These results suggest that both LO and LONs are safe for cell culture and may be used for pharmaceutical and biomedical therapies in future applications in regenerative medicine.

## 1. Introduction

Essential oils extracted from herbs, plants, and flowers are often used to manage pain, ease psychological distress, induce relaxation, and enhance well-being [1,2]. Lavender (*Lavandula angustifolia*) oil (LO) is an antispasmodic, anticonvulsant, antidepressant, painkilling, and carminative substance used to treat various conditions [3,4]. It also accelerates burn healing by modulating inflammatory reactions [5,6] and has antibacterial and antifungal properties [7]. With so many benefits, LO has been investigated as a natural medicinal extract to improve biomedical therapies and quality of life [5,6,8,9,10,11].

The field of nanotechnology has seen significant advances in numerous biomedical applications, including diagnosis and effective treatment of different disorders, tissue engineering, and regenerative medicine, using a range of nanoparticles to deliver, target, and regulate their release rate [12,13]. Recently, nanofibers of sodium alginate containing LO have been proved effective for treating induced skin injuries [5], whereas nanofibers of polyacrylonitrile containing LO were developed for antibacterial and drug delivery applications [8]. Among the different types of nanoparticles, niosomes are vesicular drug delivery systems based on the self-association of non-ionic surfactants, which can serve as a vehicle for both hydrophilic drugs (in their hydrophilic compartment) and lipophilic medicines (in their lipophilic compartment). Niosomes are biocompatible, biodegradable, non-immunogenic, and non-toxic. They can also be used in targeted delivery applications to uptake the drugs to specific sites. Furthermore, the ease of large-scale manufacturing and storage, improved stability, and healthy yet low-cost components have made niosomes an appealing alternative to conventional micro- and nano-encapsulation methods [14,15,16,17,18,19].

Adipose tissue-derived stem cells (ASCs) are characterized as mesenchymal stem cells [20]. They are used in cell therapies and regenerative medicine because of their high capacity for proliferation and their proangiogenic, antiapoptotic, and immunomodulatory properties, and are studied to treat or reduce ischemic damage in tissues [20,21,22,23,24]. Myometrial cells, in turn, represent a myofibroblast cell population involved in wound healing, extracellular matrix remodeling, and fibrosis development [25,26,27]. They can also be used as a tool to investigate the mechanisms of contractile protein synthesis and its hormone control [28].

Considering the benefits of LO and the advantages of niosomes, the aim of this study was to evaluate the toxicity of LO and LO niosomes (LONs) on ASCs and myometrial cells cultured in vitro as models for mesenchymal cells and myofibroblasts.

## 2. Materials and Methods

### 2.1. Niosomal Lavender Oil Preparation Method

LONs were prepared by a reverse-phase evaporation method (REV), one of the most common methods for preparing nanovesicular systems used for the loading of various kinds of agents, including lipids, hormones, and proteins [14,29,30]. Briefly, Span60 (S7010; Sigma, Bornem, Belgium) and cholesterol (C8667; Sigma) in a 1:1 molar ratio (50.32 mg and 48.39 mg, respectively) were mixed into 5 mL of chloroform and methanol in a 9:1 volume ratio. Fifty mg of essential oil (61718; Sigma) was then added to the mixture. Following that, 3 mL of phosphate-buffered saline (PBS; Gibco, Paisley, the Netherlands) was added, mixed by vortex mixer at room temperature for one minute, and sonicated for 5 min in a Sonicator bath (Elma^®^, Singen, Germany) at 10 °C. Elimination of organic solvent was achieved using a rotary evaporator (BUCHI R-3, Flawil, Switzerland) with the water bath temperature maintained at 65 °C, which is above the phase transition temperature of Span60 (~53 °C) and boiling point of chloroform and methanol (~62 °C). It takes around 40 min to eliminate all the solvents using rotary evaporation.

### 2.2. LON Characterization

LON vesicle size, polydispersity index and zeta potential were investigated using the dynamic light scattering (DLS) technique through the Zetasizer Nano ZS (Malvern Instruments Limited, Worcestershire, UK) [31].

### 2.3. Loading Efficiency and Loading Capacity of Lavender Oil

The loading efficiency (LE%) and loading capacity (LC%) of lavender oil in the niosomes were determined using a method described previously [32,33]. The final suspension was centrifuged (12,000× *g*, 15 min, 4 °C). The LO concentration was measured using a UV-Vis spectrophotometer at 340 nm (Thermo Fisher Scientific NanoDrop 2000/2000c, Merelbeke, Belgium). All the characterization tests were performed in triplicate. To compute the LE% and LC%, the following equations were applied (Equations (1) and (2)):

(1) $$LE\%=\frac{\left(\mathrm{WT}-\mathrm{WF}\right)\times 100\%}{\mathrm{WT}}$$

(2) $$LC\%=\frac{\left(50-W_{U.\mathrm{LO}}\right)\times 100\%}{W_{N}+50-W_{U.\mathrm{LO}}}$$

where WF is the unloaded quantity of LO detected in the supernatant after centrifugation and WT is the total amount of LO in the final niosomal suspensions. In the LC% equation, 50 (mg) is the initial amount of LO, *W*_U.LO_ is the weight of unloaded LO, and *W*_N_ is the dried weight of the niosomes.

### 2.4. LON Storage Stability Study

The LON suspension was stored at −20 °C and its DLS characteristics, as well as its LE%, were analyzed after 15 months of storage and compared with the freshly synthesized LON’s characterization. Three replicate experiments were performed. The storage stability test was performed in duplicate.

### 2.5. LON Release Study

The final LON suspension was centrifuged, and the pellet was redistributed in 5 mL culture medium, before being incubated at 37 °C. Released LO was measured at 0, 1, 2, 4, 24, and 48 h. Each time, 200 µL of the suspension was extracted and centrifuged (12,000× *g*, 15 min, 4 °C), and LO concentrations in the supernatant were calculated by spectrophotometry. The LON release study was performed in triplicate.

### 2.6. Cell Culture

ASCs from human donors were commercially acquired (Stempro^®^ Human ASCs, Thermo Fisher Scientific, Waltham, MA, USA) and characterized as previously described [21]. Myometrial cells were isolated from female donors after obtaining approval from the Institutional Review Board of the Université Catholique de Louvain (IRB reference 2020/14AOU/410). To this end, donor myometrial tissue was cut into 1 mm^2^ pieces using a sterile scalpel, then immersed in Dulbecco’s modified Eagle’s medium (DMEM/F12; 11320-033; Gibco), 1 µg/mL collagenase (C2674; Sigma) and 1 IU/µL DNase (D4263, Sigma). After incubation for 3 h in a warm bath at 37 °C with gentle agitation, the undigested tissue was filtered through a 100 µm cell strainer [34,35].

ASCs and myometrial cells were cultured in a humidified incubator at 37 °C and 5% CO_2_ in 75 cm^2^ flasks in a basic culture medium composed of DMEM/F12 1×, 10% heat-inactivated fetal bovine serum (16140071; Gibco), and 1% antibiotic-antimycotic (15240-062; Gibco). The medium was changed every other day until 80–90% confluence was reached. All cells used were between passages 6 and 8. All the in vitro experiments were performed in triplicate.

### 2.7. Cell Viability

After reaching confluence, the cells were detached using Accutase (A6964; Sigma), centrifuged at 500× *g* for 5 min at 4 °C, and resuspended in a culture medium to achieve a cell density of 100,000 cells/mL. A volume of 100 µL of each cell suspension type was added to 96-well plates for cell viability tests (10,000 cells/well). The cells were incubated on the plates in the humidified incubator (37 °C; 5% CO_2_) for 24 h to completely attach to their surface. The medium was then removed, the wells were washed with PBS (10010-015; Gibco), and a basic culture medium containing different concentrations of LO or LON (0, 0.016%, 0.031%, or 0.063%) [9] was added to each well. Culture medium without LO or LON was considered the control.

After 24 h of incubation in different media, cell viability was assessed using PrestoBlue^TM^ HS cell viability reagent (P50200; Invitrogen) and live/dead viability/cytotoxicity assays (L3224; Invitrogen, Belgium) based on protocols from Tromayer et al. [36] and Amorim et al. [37], respectively.

Briefly, after discarding the culture medium and washing the wells in PBS without Ca^2+^ and Mg^2+^, the PrestoBlue™ reagent was diluted 1:10 in the culture medium, and 100 µL was added to each well, with or without cells. Each plate was then incubated at 37 °C for one hour. Subsequently, absorbance was recorded at an emission wavelength of 620 nm and an excitation wavelength of 560 nm by fluorescence spectroscopy (Multilabel reader, Victor X4, Singapore). Data were normalized in Prism version 9.2.0 (GraphPad Software, San Diego, CA, USA), considering the average of control values as 100% viability and the average of PrestoBlue values with no cells as 0% viability.

Live/dead staining assays were performed after removing all culture media and washing the cells in warm PBS, before incubating them in 30 µL of ethidium homodimer-1 and calcein-AM solution in PBS at 37 °C for 30 min, protected from direct light. PBS-washed cells and live (green) and dead (red) cells were then monitored by fluorescence microscopy (Leica, Diegem, Belgium) using two different filters with a fluorescence excitation wavelength of 495 nm and emission wavelengths of 515 and 635 nm. All tests were conducted on three independent replicates.

### 2.8. Statistical Analysis

Data were statistically analyzed by one-way ANOVA using GraphPad Prism 9.2.0, and quantitative data are presented as mean ± SD. Error bars in graphs indicate one sample standard deviation.

## 3. Results

### 3.1. LON Characterization, Storage Stability, and Release Profile

DLS analysis was used to calculate LON size and the polydispersity index. Moreover, to prove the stability of LONs in term of DLS characteristics, LE%, and LC%, the same characterization tests were done 15 months after being stored at −20 °C (Table 1). There was no significant difference in the characteristics of fresh LONs and LONs stored for 15 months stored.

**Figure 1:**
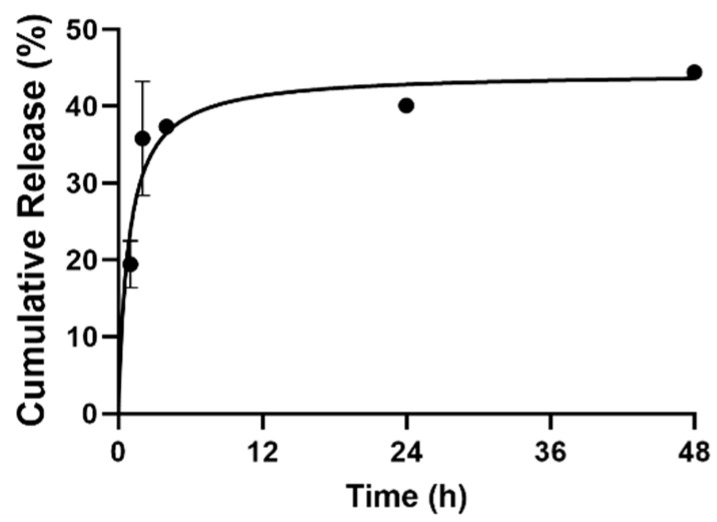
In Vitro cumulative release profile of LO from LONs over a two-day period. Graphic made with GraphPad Prism 9.0 (*n* = 3).

We also investigated LO release profiles from a LON vesicular system and found that 44.44 ± 0.62% of loaded LO was released after 48 h, whereas 37.34 ± 0.35% of that amount was released in the first 4 h of incubation (Figure 1).

### 3.2. Cytotoxicity

**Figure 2:**
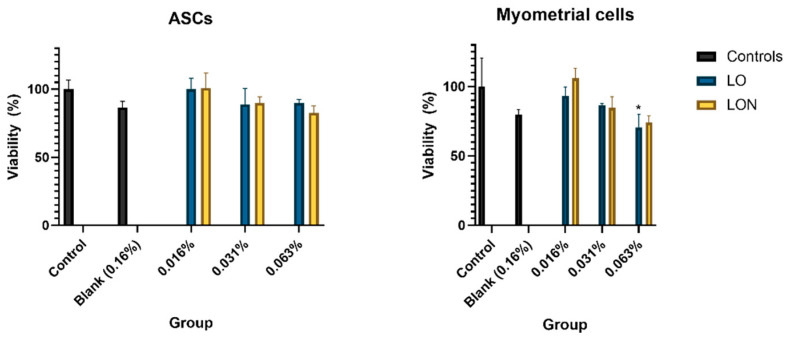
Cell viability evaluated with PrestoBlueTM for ASCs and myometrial cells cultured with and without LO and LONs. The asterisk represents a significant difference (*p* < 0.05) from controls. Graphic made with GraphPad Prism 9.0 (*n* = 3).

The viability of ASCs and myometrial cells in LO and LON media is shown in Figure 2. In ASCs, viability rates ranged from 88.77 ± 2.59% to 100.03 ± 8.05% for LO concentrations, and from 82.67 ± 5.08% to 100.78 ± 10% for LON. In myometrial cells, they ranged from 70.6 ± 9.43% to 93.23 ± 6.34% and from 74.07 ± 4.84% to 105.97 ± 7.18% for LO and LON, respectively.

**Figure 3:**
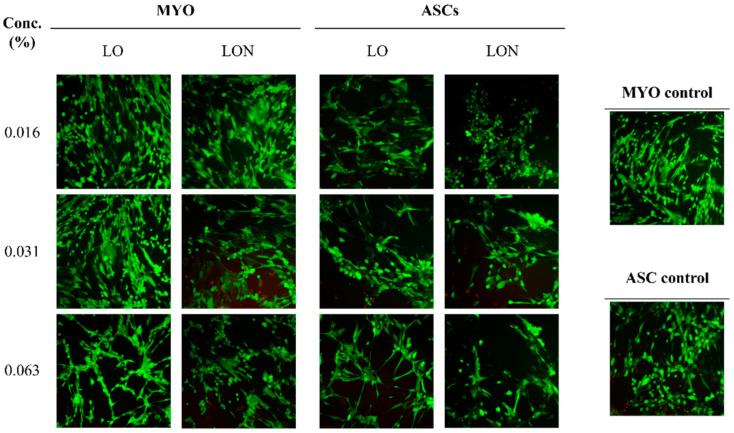
Cytotoxicity of LO and LON in different concentrations for myometrial cells (MYO) and ASCs using live/dead assays. Live cells are shown in green and dead cells in red (*n* = 3).

Values of 0.063% LO in myometrial cells were significantly lower than in controls (*p* < 0.05). Live/dead assays confirmed the low number of dead cells (Figure 3).

## 4. Discussion

Essential oils are volatile, hydrophobic, and viscous substances, making them difficult to dissolve in hydrophilic media. For this reason, they are usually diluted in Tween, dimethyl sulfoxide, or ethanol [7]. Niosomes offer a novel alternative for diluting these substances in aqueous environments since they are non-ionic surfactant vesicles used as carriers for hydrophilic and lipophilic substances, easily diluted in culture media, and non-toxic to cells. Moreover, they allow controlled release of the substance, increase its bioavailability, and enhance absorption of some drugs across cell membranes. In the present study, LON yielded cell viability similar to controls and LO in both ASCs and myometrial cells, which could prove advantageous for future applications.

The size of a vesicular drug delivery system is a key factor in its biodistribution, drug encapsulation, and pharmacokinetics [38]. LON size and polydispersity index were found to be comparable with other niosomal vesicle-loading hydrophobic agents [39]. Moreover, LON size was smaller than liposomal globulus, afra, alternifolia, and other types of essential oils encapsulated in phospholipid-based vesicular carriers [40].

Zeta potential is a helpful tool for estimating the magnitude of colloidal particle interactions and plays an important role in nanoparticle stability and biocompatibility [14,41]. LON zeta potential was −22.4 ± 0.9, which showed that the particles were negatively charged and more stable against aggregation and fusion [14]. LO LE% in niosomes was also comparable with other hydrophobic agent niosomal systems loaded with the same primary components [42]. In this study, the storage stability of LON at −20 °C was studied in terms of size, polydispersity, Zeta, and LE%; the results showed the same DLS parameters as well as no leakage of the LO. Junyaprasert et al. also studied niosomes kept at 4 °C for 3 months and reported the same DLS characteristics and LE% [43]. The resulting release profile is comparable with release rates of other hydrophobic agents loaded inside niosomes, where more than 20% of loaded silibinin was released in the first 6 h [42].

LO has shown antispasmodic properties in the ileum and uterine smooth muscle in animal studies, increasing vasodilation and relieving pain and discomfort after labor [7]. It also has antimicrobial properties, successfully treating bacterial infections resistant to antibiotics [44] and inhibiting fungal sporulation and respiration [45]. These properties can be harnessed to develop pharmaceutical products using natural products in regenerative medicine.

There was no significant difference between the viability of ASCs and myometrial cells treated with blank niosomes and untreated cells, which means the nanoparticles are biocompatible. The in vitro biocompatibility of niosomes was proved in our previous studies using different cell types [15,30,46]. Toxicity tests revealed that both LO and LON have cell viability similar to the normal culture medium, making them safe for use in cell culture. The only exception was 0.063% LO, which showed lower viability in myometrial cells. Although the viability significantly decreased in the highest dose, it still remained over 70% in both LO and LON treatments.

## 5. Conclusions

This study has demonstrated that LO and LONs are safe to use in both stem cell and differentiated cell cultures in vitro. Although LO has various beneficial effects, it may be limited by a number of drawbacks, such as volatility, hydrophobicity, and viscosity. The present study shows that loading LO into niosomal formulations can overcome these disadvantages. Indeed, this approach shows high potential for use in regenerative medicine in future pharmaceutical and biomedical applications.

## Figures and Tables

**Table 1:** The size, polydispersity index, zeta potential, loading efficiency, and loading capacity of freshly synthesized LON and after 15 months’ storage at −20 °C.

Time (Months)	Size (nm)	Polydispersity Index	Zeta Potential (mV)	LE (%)	LC (%)
0	1216 ± 106	0.328 ± 0.045	−22.4 ± 0.9	78.032 ± 2.141	28.35 ± 0.56
15	1142.5 ± 47.5	0.425 ± 0.035	−24.55 ± 1.45	73.99 ± 4.35	27.26 ± 1.17

## Data Availability

Not applicable.
